# Count Gattenberg, Member of the Lower Austrian District Committee, Celebrates the Twenty-fifth Anniversary of That Event

**Published:** 1897-10

**Authors:** 


Count Gattenberg, Member of the Lower Austrian District Committee, Celebrates the Twenty-fifth Anniversary of that Event.—Constantine, Count of Gattenberg, for many years an honorary member of the Veterinary Association in Austria, on September 25th celebrated his twenty-fifth annual jubilee as member of the Lower Austrian District Committee. Inasmuch as Count Gattenberg had given so many proofs of his recognition of the efforts of veterinarians, and the veterinary organization of Lower Austria had received under his direction a very high development, the association of veterinarians in Austria sent a deputation, consisting of President Toscano, Secretary Dauscher, and Central Committeeman Wittmann, to congratulate him in the name of the association. On October 3d the officials and veterinary committee presented the Count with a beautiful album containing their photographs. Certificates of honorary membership in many associations were handed to the Count. At the banquet in the Hotel Elizabeth twelve veterinarians were present. District Veterinarian Dr. Saass proposed a very appropriate toast in honor of the Count.—*Idem.*

